# Development and Competition of Three Parasitoid Wasps, *Brachymeria podagrica, Dirhinus himalayanus*, and *Nasonia vitripennis*, in Their Host, *Sarcophaga dux*, in Single and Mixed Infections

**DOI:** 10.3390/pathogens13070572

**Published:** 2024-07-09

**Authors:** Rolf K. Schuster, Saritha Sivakumar

**Affiliations:** Central Veterinary Research Laboratory, Dubai P.O. Box 597, United Arab Emirates; saritha@cvrl.ae

**Keywords:** parasitoid wasps, *Brachymeria podagrica*, *Dirhinus himalayanus*, *Nasonia vitripennis*, *Sarcophaga dux*

## Abstract

Laboratory trials were carried out to investigate the development of three entomophagous parasitoid wasps in preimaginal stages of *Sarcophaga dux* in monoinfections and mixed infections. Laboratory-raised postfeeding *S. dux* third-stage larvae were exposed to *Brachymeria podagrica*. After pupation, 50 of these fly puparia were brought in contact with pupal parasitoid *Dirhinus himalayanus* and 50 with *Nasonia vitripennis*, and the remaining 50 puparia were left as *Brachymeria* monoinfection. In three further trials, each set of 50 freshly pupated host puparia from the same source was exposed to *N. vitripennis* and *D. himalayanus*, as monoinfections and mixed infections, respectively. The uninfected control group consisted of 50 *S. dux* larvae that were kept separately under the same conditions. The percentages of successfully developed *B. podagrica* and *D. himalayanus* in monoinfections were 56 and 86%, respectively, and progeny of *N. vitripennis* hatched from 88% of the exposed host puparia. In mixed infections, *N. vitripennis* dominated over *B. podagrica* and *D. himalayanus* with rates of successfully infected hosts of 50 and 94%, respectively. The number of *Nasonia* progeny in these groups ranged from 4 to 49 and 5 to 43, respectively. *Dirhinus himalayanus* did not develop in the simultaneous infection with *N. vitripennis*. Not a single *S. dux* eclosed in the six experimental groups, while in the uninfected control group, 46 (92%) adult flies eclosed 11 to 14 days after the start of pupation. Since the three parasitoids emerge from flesh fly pupae, these insects can become important in criminal forensic investigations when corpses are in an advanced stage of decay. More data on their preimaginal development at different temperatures are necessary.

## 1. Introduction

The smell of a decaying carcass attracts a variety of insects that are used in forensic entomological examinations to determine the postmortem interval. One of these insects is *Sarcophaga (Liosarcophaga) dux* Thomson, 1896, a necrophagous fly belonging to the Sarcophagidae family. As a facultative myiasis parasite, this species is also of medical importance [1,2,3]. Geographically, *S. dux* occurs in many part of the world including the Indomalayan realm [1], the Middle East [4], and western Europe [5]. Since this fly species is attracted to carcasses, it has also an importance in criminal forensic investigations [6,7,8,9].

In addition to necrophorous insects, certain parasitoid wasps also seek proximity to carcasses aiming to deposit their eggs in fly larvae or pupae [10]. For this reason, parasitoid wasps also can have an importance in forensic entomology [11,12,13], but in practice, their biology is rarely applied to criminal investigations. This is because of their small size and the tendency to arrive during later stages of decay [14].

In order to determine the length of the preimaginal development of sarcophagid flies during the hot summer months in the United Arab Emirates (UAE), the presence of two parasitoids, *Brachymeria podagrica* (Fabricius, 1787) and *Dirhinus himalayanus* Westwood, 1836, of the Chalcididae family were detected in August 2021 [15]. These species were already previously mentioned in the insect fauna of the UAE [16]. Both *B. podagrica* and *D. himalayanus* are solitary idiobionts since they develop in non-growing host stages. While the endoparasitic *B. podagrica* injects its eggs into the soft tissues of third-stage larvae of muscoid flies, the ectoparasitic *D. himalayanus* deposits its eggs in the fly puparium on the developing host pupa. Although multiple eggs and early larval stages can be found, as a result of intraspecific competition, only a single wasp will undergo a full development.

A third parasitoid, *Nasonia vitripennis* (Walker, 1836), was detected in naturally infected *S. dux* puparia in May 2022. This species is a tiny representative of the Pteromalidae family that has a world-wide distribution [17] and is mentioned here as a new species for the UAE fauna for the first time. Like *D. himalayanus*, *N. vitripennis* is an ectoparasitoid depositing its eggs on fly pupae within a puparial shell. But the successful development results in multiple *Nasonia* progeny.

All three wasp species are parasitoids of filth flies and were maintained for several generations on preimaginal stages of the flesh fly, *S. dux*. In multiple laboratory infection trials carried out at a constant temperature of 26 °C, the preimaginal development of *N. vitripennis, B. podagrica*, and *D. himalayanus* lasted 15–17, 20–28, and 29–37 days, respectively.

So far, these trials were carried out as monoinfections, but under natural conditions, fly development stages are exposed to different parasitoids, and for this reason, it is of practical importance to know the outcome of mixed infections and which of the three entomophagous wasp species dominate over others. 

## 2. Materials and Methods

In order to produce a sufficient number of preimaginal host development stages, 500 g of camel meat matured for two days at room temperature was kept for three days in a plastic bowl in a cage containing a laboratory colony of *S. dux*. The temperature in the fly cage varied between 30 and 32 °C, and the light regime was 12:12 h light–dark cycle. With the occurrence of third-stage larvae, the bowl with the meat sample was removed and placed into a larger bucket with a layer of 3 cm fine-grained sand. More than 400 postfeeding fly larvae left the decaying meat and were sieved out from the sand on the fifth day of larval development. Of these, 350 larvae were used for this experiment. 

In a first step, 150 maggots were transferred in a 20 L aquarium glass with a 1 cm layer of fine-grained dry sand and were exposed for 24 h to 15 mated female *B. podagrica* on day 5. Each set of 50 larvae was then transferred into Petri dishes. After pupation, three experimental groups were formed: group B (*Brachymeria* monoinfection), group BD (*Brachymeria-Dirhinus* superinfection), and group BN (*Brachymeria-Nasonia* superinfection). For the superinfections, 10 mated *D. himalayanus* and 10 mated *N. vitripennis* females were added on day 9 to the subsequent groups (Table 1). 

Another 200 fresh postfeeding larvae from the same source were equally distributed into four Petri dishes and were allowed to pupate. Each set of 50 pupae was used as hosts for the pupal parasitoids *D. himalayanus* and *N. vitripennis* to form three further experimental groups: group D (*Dirhinus* monoinfection), group N (*Nasonia* monoinfection), and group DN (*Dirhinus-Nasonia* mixed infection) on day 8. The uninfected control group consisted of another 50 pupae. (Table 1). 

*D. himalayanus* and *N. vitripennis* were allowed to stay in contact with the fly pupae for 10 days. In all experimental groups, the relation of preimaginal fly development stages to fertile female parasitoids was 10:1. All the trials were carried out in a room with a constant temperature of 26 °C. 

In the experimental groups B, D, and BD, the number of eclosed puparia and the sex of the emerged wasps were recorded at daily inspections. Unhatched puparia were opened, and their content (dry or brown pasty content, dead flies or dead wasps unable to hatch including preimaginal wasp development stages) was recorded.

Those groups where gregarious *N. vitripennis* were involved (N, DN, and BN) were treated differently in order to be able to count the progeny. As it was known from other references [14,18] and from our own multiple pretrials, the preimaginal development for *N. vitripennis* at 26 °C lasted 15–17 days. Thus, individual puparia of the three groups N, DN, and BN were placed into small Petri dishes (35 mm diameter) 10 days after the start of the *Nasonia* infection. In those groups, the experiments were terminated 18 days after the addition of the wasps by keeping the Petri dishes at −20 °C for 48 h in the deep freezer. The number of emerged wasp progeny was counted, and the content of uneclosed puparia was examined under the stereo microscope. 

### Statistical Treatment

The software Quantitative Parasitology (QPweb) (Version 3.0) [19] was used to generate the prevalence, intensity, and abundance of *N. vitripennis* in the groups N, DN, and BN including the subsequent 95% confidence intervals.

## 3. Results 

Right at the start of the infection trial for about 30 min., *B. podagrica* females behaved neutral to fly larvae. This behavior changed after single wasps started to attack the maggots. The wasps crawled on their hosts and inserted their ovipositor through the skin of the fly larvae. Often, they also crawled backward toward a nearby maggot and stung it. The contact between wasp and maggot lasted only few seconds since the fly larvae tried to dislodge the parasitoid. Successfully attacked maggots excreted a small amount of a smelly liquid. No special selection neither to a special maggot nor to a special body site was observed.

The other two wasp species, *D. himalayanus* and *N. vitripennis*, carefully observed fly pupae by “drumming” with their antennae on the puparial shell. When a suitable place was found, they pricked an opening into the puparial shell by a sawing movement of their ovipositor, to deposit their eggs.

As seen from Table 1, pupation in the groups D, N, and ND, as well as in the uninfected control group, started on day 6 and was completed on day 8, while in the initially *Brachymeria*-infected groups (B, BD, and BN), pupation was retarded and was completed between day 9 and day 11 only. Out of the 350 used hosts, a total of four third-stage larvae did not develop and dried out prior to pupation. 

### 3.1. Uninfected Control Group

**Figure 1:**
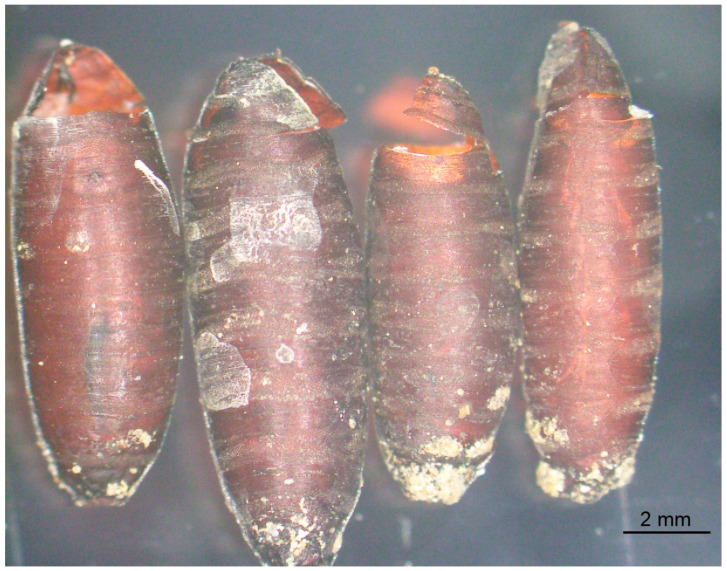
Empty puparial shell of *Sarcophaga dux* after fly imagos eclosed.

In the uninfected control group, 46 (92%) *S. dux* emerged between day 17 and 20 days after the meat bait was placed in the fly cage, 11 to 14 days after pupation. One maggot did not pupate and dried out. The dissection of the three uneclosed puparia revealed two dead flies unable to hatch, and one puparium contained a smelly brown pasty content (Table 2). Filth flies always emerged from the anterior side of the puparium by breaking the upper part. In the majority of cases the upper part of the puparium was still connected to the rest (Figure 1).

### 3.2. Group B

**Figure 2:**
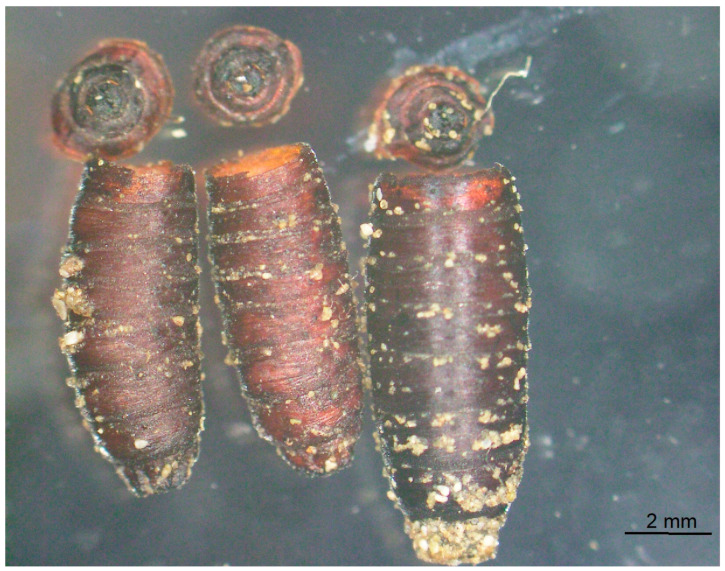
Empty puparial shell of *Sarcophaga dux* after *Brachymeria podagrica* eclosed. The lid of the puparium was separated from the rest of the puparial shell.

Two maggots of group B did not pupate and dried out. A total of 28 (56.0%) *B. podadica* (24 males and 4 females; male–female ratio of 6:1) emerged between day 20 and day 25 after infection. Of 20 uneclosed fly puparia, 16 had a dry content, and each one contained brown sticky masses, a dead wasp pupa, a dead female *B. podagrica*, and a dead fly unable to hatch (Table 2). Also, *B. podagrica* hatched through the anterior part of the host puparium after the wasp had nibbled the puparial shell along the line of weakness. As a result, the lid was separated from the rest of the puparium in most cases (Figure 2). The puparium was separated from the remaining puparial shell.

### 3.3. Group BD

A total of 28 parasitoid wasps emerged in this group. These were 18 male and 6 female (male–female ratio of 3:1) *B. podagrica* that eclosed 20 to 24 d after fly larvae were exposed. Two males and two females of *D. himalayanus* hatched between day 33 and day 38 after the first contact of the wasps with their host pupae. Most of the remaining puparia had a dry content, and four of them contained dead *Brachymeria* pupae, while in two further puparia, dead *B. podagrica* imagos were seen (Table 2).

### 3.4. Group N

**Figure 3:**
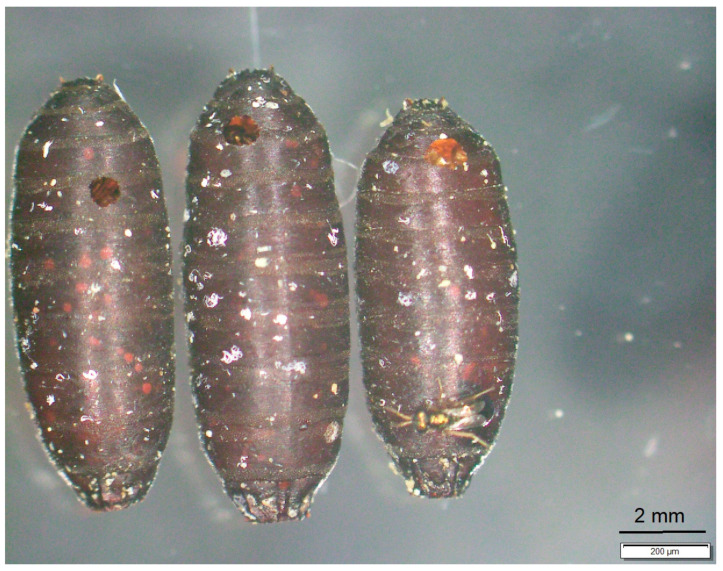
Empty puparial shell of *Sarcophaga dux* after *Nasonia vitripennis* eclosed through small holes at the anterior end of the puparium.

Between day 15 and day 18, *N. vitripennis* adults hatched from 44 (88%) puparia. The number of progeny per infected host puparium ranged between 2 and 45 (total: 1208; average: 27.5). Dry or brown pasty content was observed in one and five uneclosed puparia, respectively. *N. vitripennis* emerged through a round hole of around 1 mm in diameter. In most cases, this loophole was situated at the anterior part, less frequently in the middle or at the posterior parts of the puparium (Figure 3).

### 3.5. Group DN

In total, 47 (94.0%) host puparia were infected with *N. vitripennis*. A total of 1090 *Nasonia* progeny with a range between 5 and 43 (average 23.2) eclosed between day 15 and day 17. Of the three remaining puparia, two had a dry content, and one was filled with brown pasty masses. In this group, there were no signs of *D. himalayanus* development (Table 3 and Table 4). 

### 3.6. Group BN

One of the originally used maggots did not pupate and dried out. Four male and one female *B. podagrica* hatched 20 and 21 d after *Brachymeria* infection, and four further puparia contained late *Brachymeria* pupal stages. A total of 25 fly puparia produced a total of 480 *Nasonia* progeny (average: 19.2; range 4–49). This was significantly lower than in the groups N and DN. A dry content was found in 15 uneclosed puparia (Table 3 and Table 4). 

**Figure 4:**
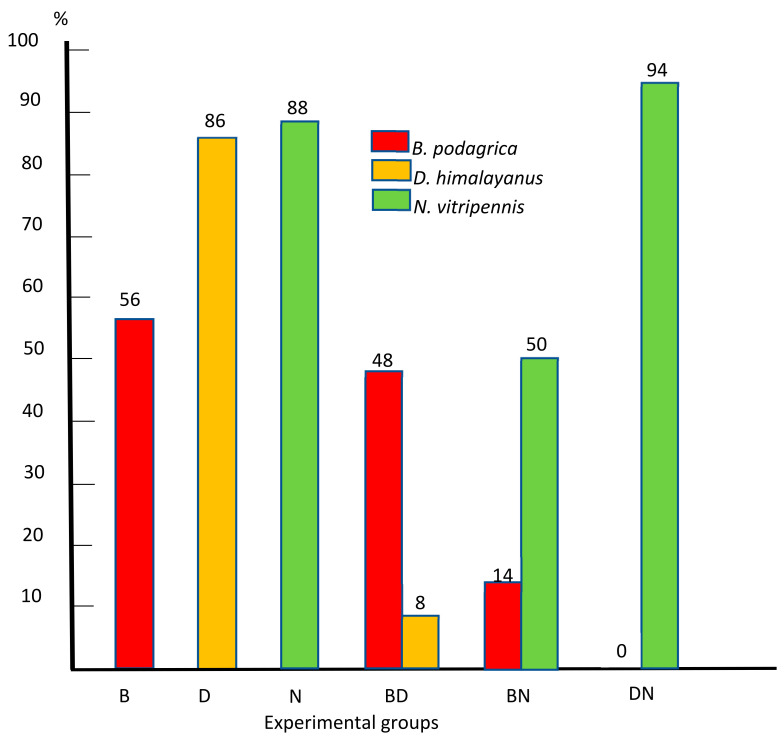
Percentage of successfully infected preimaginal stages of *S. dux* in monoinfections with *Brachymeria podagrica* (B), *Dirhinus himalayanus* (D), and *Nasonia vitripennis* (N) and mixed infections with *Brachymeria podagrica* + *Dirhinus himalayanus* (BD)*, Brachymeria podagrica* + *Nasonia vitripennis* (BN), and *Dirhinus himalayanus* + *Nasonia vitripennis* (DN).

Figure 4 summarizes the percentage of successfully infected hosts in all six experimental groups.

## 4. Discussion

In our trials, already two days after the postfeeding sarcophagid larvae had left the meat sample, pupation started and was completed within the next days, except for the experimental groups where *B. podagrica* was involved. Since *B. podagrica* could only infect fly larvae, the time window for the infection was very short, and for this reason, the female wasp had to act quickly. The attack of the maggots by *B. podagrica* and the subsequent inoculation of its eggs into the host caused stress, and this might be the reason for a delayed pupation.

With the inoculation of its eggs into the host, the parasitoid wasps injected also a venom in order to paralyze the host, to arrest its development, or to evade host defense mechanisms. The biological functions of such venoms were reviewed by Moreau and Asgari [20]. For the endoparasitoid *B. podagrica* the venom has not yet been studied, and in our experiments it did not have an obvious paralytic effect, but it might have an action on the host’s immune system preventing the encapsulation of the wasp’s eggs and early larval stages. As it was already noticed in other trials, multiple assaults on a single maggot are feasible and might lead to possible envenomization and eventually to the death of the host in an early stage of pupation [21]. On the other hand, contrary to the other two wasp species, *B. podagrica* deposits its eggs deep into the soft tissues of the maggot and allows the host to undergo pupation. With the ovipositor that penetrates the cuticle of the fly larva, *B. podagrica* might inject other pathogens, and this also can lead to the death of the maggot before it pupates.

In any case, even after successful pupation, the development of the fly within the puparial shell can be terminated as it was seen in the experimental groups B, BD, and BN in which 17/48, 16/50, and 15/49 of the uneclosed puparia were dry or had a brown pasty content. The percentage of dead fly pupae in the groups without *Brachymeria* involvement (groups D, N, and DN) was considerably lower and equaled 5/50, 6/50, and 3/50, respectively.

Although multiple *Brachymeria* eggs and early larval stages can be found after infection, as a result if intraspecific (ciblicide) competition, only one wasp will successfully undergo a full development [21]. As it is known for other solitary parasitoids, the mandibulate first instars behave aggressively toward potential competitors [22,23].

Females of *D. himalayanus* and *N. vitripennis* lay their eggs in the space between the inner puparial wall and the developing fly. Once these wasps have located the host, they spend some time to examine the puparium to make an oviposition decision. For this, they use their antennae in drumming movements or persevere quietly to receive vibrations in order to obtain information on quality of the content. The venom that is injected at oviposition arrests the development of the fly within the host purarium, and in our pretrials it was observed that *N. vitripennis* underwent successful development even when *S. dux* was already in a late pharate stage, shortly before eclosure. In detail, the action of the venom is known only for *N. vitripennis* [24,25,26], and the nature of the venom of *D. himalayanus* has not been examined yet but may have a similar effect.

Since in the DN group there was no sign of a development of *D. himalayanus*, it is imaginable that due to the more quickly developing superior numbers of *Nasonia* larvae, there was not enough food for the competitor of the other species, and the much more slowly developing *Dirhinus* larval stage might have starved to death.

While *B. podagrica* and *D. himalayanus* are solitary parasitoids that produce only one offspring per host, the gregarious *N. vitripennis* was the most successful species in terms of numbers of broods. The average number of offspring in the groups N and DN equaled 24.4 and 21.8, respectively, and the maximum number of progeny in these groups was 45 and 43, respectively.

Contrary to this, the *Nasonia* brood size in the group BN was considerably less and equaled on average 9.6 in only 25 *Nasonia* infected puparia. The reason for this might be that at the time of *Nasonia* infection, *Brachymeria* larvae were already four to five days old. At this age, *Brachymeria* larvae had reached their second stage and had consumed already a substantial part of the host’s body, leaving fewer resources for the developing *Nasonia* larvae. It is possible that *N. vitripennis* females recognized that these hosts were not suitable for an infection. There was also a high number (n = 15) of host puparia with a dry content without any visible wasp development. This could have been the result of multiple *Brachymeria* attacks and subsequent envenomization at the beginning of the trial.

In the *D. himalayanus* monoinfection (group D), 43 wasps emerged from 50 offered pupae, and a further wasp died in a late pupal stage. This high potential of *D. himalayanus* in monoinfections was already observed in a previous experimental study where 90 and 98% of the used parasitoids developed in *S. dux* and *W. nuba* pupae, respectively [15]. In the mixed infection of group BD, *D. himalayanus* was less successful. In this group, only four *D. himalayanus* emerged compared with 24 *B. podagrica*. Two possible reasons can be considered. Females might have recognized that the puparia were already occupied, and there were not enough host resources, or the mandibulate *Brachymeria* larvae might have killed the competitor as a result of interspecific competition.

Since all three wasp species parasitize preimaginal stages of necrophageous fly development stages, larval and pupal stages of these parasitoids can be found in fly puparia next to decaying carcasses. In order to make use of these parasitoids, their life cycle has to be studied under various temperature conditions as was performed already for *N. vitripennis* [11,18]. In our experiments at 26 °C, *N. vitripennis* with a preimaginal development duration of 15 to 17 days started to emerge two days after the last fly in the control group had eclosed. The development of *B. podagrica* started with the inoculation of its eggs into the third-stage fly larvae.

The time slot for oviposition for this wasp was short because already on day four, fly larvae started to leave the meat sample searching for a shelter for pupation. With the start of the pupation, the possibility of an infection ends. The development of *B. podagrica* lasted 20 to 25 days. Thus, the first wasps of this species hatched four days after *S. dux* in the control group. *D. himalayanus* could be of special interest since its development is considerably longer than that of the fly host, and wasp development stages are still present when uninfected host flies have eclosed already, leaving empty puparial shells behind.

Even empty puparial shells can tell something about the organism that has emerged. At emergence, sarcophagid and other muscoid flies inflate a membranous sac (ptilinum) that protrudes from the face above the antennae, and due to this pressure the puparium breaks at the line of weakness in the anterior part the puparium. The upper part of the empty puparium is still connected. Both chalcidid wasps also emerge from the anterior of the puparium but in a different way. They nibble the puparial shell from inside at the anterior fifth. In the case of *B. podagrica*, very often the upper part of the puparium was separated during the emergence of these wasps. Contrary to this, pteromalid wasps leave the puparium through a small round hole that they nibble in the puparial shell.

## 5. Conclusions

These experiments showed that all three wasp species were effective in preventing the development of their host species *S. dux* in all six infection trials. Taking the number of offspring as a criterion, *N. vitripennis* was the most successful species. Its development in the host puparium under laboratory conditions at 26 °C lasted only 15 to 17 days, while for the preimaginal development of *B. podagrica* and *D. himalayanus* it took 20–25 and 31–38 days, respectively. In mixed infections, *N. vitripennis* suppressed the development of *D. himalayanus* but did not fully inhibit the development of *B. podagrica*. Most probably, females of *N. vitripennis* recognized that some of the offered puparia were already preinfected. In order to make use of parasitoid wasps for forensic research, their life cycles need to be studied under different temperature regimes.

## Figures and Tables

**Table 1:** Cumulative number of pupated maggots and day of addition of parasitoids (^1^: *B. podagrica*; ^2^: *D. himalayanus*; ^3^: *N. vitripennis*) to the experimental groups (B: *Brachymeria* monoinfection; BD: *Brachymeria Dirhinus* superinfection; BN: *Brachymeria Nasonia* superinfection; D: *Dirhinus* monoinfection; N: *Nasonia* monoinfection; DN: *Dirhinus Nasonia* mixed infection).

Days	Experimental Groups						
	B (n = 50)	BD (n = 50)	BN (n = 50)	D (n = 50)	N (n = 50)	DN (n = 50)	Control (n = 50)
5	0 ^1^	0 ^1^	0 ^1^	0	0	0	0
6	0	0	0	6	5	2	3
7	12	8	8	42	44	37	41
8	40	39	39	50 ^2^	50 ^3^	50 ^2,3^	49
9	47	49 ^2^	49 ^3^	50	50	50	49
10	47	50	49	50	50	50	49
11	48	50	49	50	50	50	49
No. of dead L3	2	0	1	0	0	0	1

**Table 2:** Infection success (number of eclosed insects) in monoinfection with solitary parasitoid wasps, *B. podagrica* (B) and *D. himalayanus* (D), and mixed infection of both parasitoids (BD) in comparison with the uninfected control group (Control). (* *B. podagrica*.).

		Experimental Groups			
		B	D	BD	Control
Eclosed	*B. podagrica*	28	-	24	-
	*D. himalayanus*	-	43	4	-
	*S. dux*	0	0	0	46
	**Total eclosed:**	**28**	**43**	**28**	**46**
Uneclosed puparia with	Dry content	16	3	16	0
	Brown pasty content	1	2	0	1
	Dead wasp larvae/pupae	1	1	4	-
	Dead wasps unable to hatch	1	0	2 *	-
	Dead flies unable to hatch Dried fly L3	1 2	1 0	0 0	2 1
	**Total uneclosed puparia:**	**22**	**7**	**22**	**4**

**Table 3:** Number of *Sarcophaga* pupae that were infected with parasitoid wasps in three experimental groups and content of dead fly puparia (N: *N. vitripennis*; DN: *D. himalayanus* and *N. vitripennis*; BN: *B. podagrica* and *N. vitripennis*).

Wasp Species	Experimental Groups		
	N	DN	BN
*N. vitripennis*	44	47	25
*D. himalayanus*	-	0	-
*B. podagrica*	-	-	5
Uneclosed puparia with *Brachymeria* pupae	-	-	4
Dry content	1	2	15
Brown pasty content	5	1	0

**Table 4:** Development of *N. vitripennis* in a monoinfection and in superinfections with *D. himalayanus* and *B. podagrica.* Confidence intervals shown in brackets.

	*N. vitripennis* Progeny in Three Experimental Groups		
	N	DN	BN
Number of infected hosts puparia	44	47	25
Percentage of infected (%)	88.0 [75.7–95.5]	94.0 [83.5–98.7]	50.0 [35.5–64.5]
Average intensity	27.5 [23.5–30.3]	23.2 [21.1–25.1]	19.2 [15.1–24.5]
Average abundance	24.4 [20.0–27.9]	21.8 [19.2–24.1]	9.6 [6.5–14.0]

## Data Availability

The original data are available on request.
